# Hemolysis during cardiac surgery is associated with increased intravascular nitric oxide consumption and perioperative kidney and intestinal tissue damage

**DOI:** 10.3389/fphys.2014.00340

**Published:** 2014-09-08

**Authors:** Iris C. Vermeulen Windsant, Norbert C. J. de Wit, Jonas T. C. Sertorio, Annemarie A. van Bijnen, Yuri M. Ganushchak, John H. Heijmans, Jose E. Tanus-Santos, Michael J. Jacobs, Jos G. Maessen, Wim A. Buurman

**Affiliations:** ^1^Department of Surgery, Maastricht University Medical CenterMaastricht, Netherlands; ^2^NUTRIM School for Nutrition, Toxicology & Metabolism, Maastricht University Medical CenterMaastricht, Netherlands; ^3^Central Diagnostic Laboratory, Maastricht University Medical CenterMaastricht, Netherlands; ^4^Department of Pharmacology, Ribeirao Preto Medical School, University of Sao PaoloRibeirao Preto, Brazil; ^5^Department of Extracorporeal Circulation, Maastricht University Medical CenterMaastricht, Netherlands; ^6^Department of Anesthesiology, Maastricht University Medical CenterMaastricht, Netherlands; ^7^Cardiovascular Research Institute Maastricht, Maastricht University Medical CenterMaastricht, Netherlands; ^8^Department of Vascular Surgery, European Vascular Center Aachen-Maastricht, University Hospital AachenAachen, Germany; ^9^Department of Cardiothoracic Surgery, Maastricht University Medical CenterMaastricht, Netherlands

**Keywords:** hemolysis, cardiopulmonary bypass, acute kidney injury, nitric oxide, intestinal fatty acid binding protein

## Abstract

**Introduction:** Acute kidney injury (AKI) and intestinal injury negatively impact patient outcome after cardiac surgery. Enhanced nitric oxide (NO) consumption due to intraoperative intravascular hemolysis, may play an important role in this setting. This study investigated the impact of hemolysis on plasma NO consumption, AKI, and intestinal tissue damage, after cardiac surgery.

**Methods:** Hemolysis (by plasma extracellular (free) hemoglobin; fHb), plasma NO-consumption, plasma fHb-binding capacity by haptoglobin (Hp), renal tubular injury (using urinary N-Acetyl-β-D-glucosaminidase; NAG), intestinal mucosal injury (through plasma intestinal fatty acid binding protein; IFABP), and AKI were studied in patients undergoing off-pump cardiac surgery (OPCAB, *N* = 7), on-pump coronary artery bypass grafting (CABG, *N* = 30), or combined CABG and valve surgery (CABG+Valve, *N* = 30).

**Results:** FHb plasma levels and NO-consumption significantly increased, while plasma Hp concentrations significantly decreased in CABG and CABG+Valve patients (*p* < 0.0001) during surgery. The extent of hemolysis and NO-consumption correlated significantly (*r*^2^ = 0.75, *p* < 0.0001). Also, NAG and IFABP increased in both groups (*p* < 0.0001, and *p* < 0.001, respectively), and both were significantly associated with hemolysis (*R*_s_ = 0.70, *p* < 0.0001, and *R*_s_ = 0.26, *p* = 0.04, respectively) and NO-consumption (*R_s_ = 0.55, p* = 0.002, and *R*_s_ = 0.41, *p* = 0.03, respectively), also after multivariable logistic regression analysis. OPCAB patients did not show increased fHb, NO-consumption, NAG, or IFABP levels. Patients suffering from AKI (*N* = 9, 13.4%) displayed significantly higher fHb and NAG levels already during surgery compared to non-AKI patients.

**Conclusions:** Hemolysis appears to be an important contributor to postoperative kidney injury and intestinal mucosal damage, potentially by limiting NO-bioavailability. This observation offers a novel diagnostic and therapeutic target to improve patient outcome after cardiothoracic surgery.

## Introduction

Visceral complications significantly contribute to increased morbidity and mortality after cardiac surgery, and lengthen duration of stay at the intensive care unit (ICU) and total hospitalization time. More specifically, loss of intestinal wall integrity, for instance due to intestinal ischemia, promotes bacterial translocation, and may induce a systemic inflammatory response (SIRS) or sepsis (Grotz et al., 1999; Huybregts et al., 2007). Acute kidney injury (AKI) is also common (5–30%) (Conlon et al., 1999; Lassnigg et al., 2004) and associated with high morbidity and mortality, particularly when patients require dialysis (Rosner and Okusa, 2006).

The identification of potentially modifiable risk factors for the development of visceral injury during cardiac surgery is imperative for the development of specific treatment strategies to improve patient outcome. The use of cardiopulmonary bypass (CPB) significantly contributes to the development of intestinal mucosal injury and renal tubular damage through induction of (microcirculatory) blood flow alterations, ischemia-reperfusion injury, hemodilution, and a pro-inflammatory response (Kameneva et al., 1999; Loef et al., 2002; Schrier et al., 2004). Another common consequence of CPB use is the development of intravascular hemolysis characterized by an acute rise of circulatory cell free hemoglobin (fHb) (Vercaemst, 2008). The discovery of the potent nitric oxide (NO) scavenging property of fHb has been associated with decreased microcirculatory NO-bioavailability, decreased organ perfusion and renal function, and increased mortality during exacerbations of diseases characterized by *chronic* hemolysis (Reiter et al., 2002; Rother et al., 2005). Recently, we have shown that also *transient* increases in plasma fHb concentrations are independently associated with proximal renal tubular injury and postoperative AKI after CPB-assisted thoracoabdominal aortic surgery (Vermeulen Windsant et al., 2010).

In the present study we tested the hypothesis that hemolysis during cardiac surgery is (1) principally a consequence of CPB, (2) leads to increased intravascular consumption of NO (reflecting decreased NO-bioavailability, Reiter et al., 2002), and (3) contributes to the development of visceral tissue damage and postoperative renal dysfunction. In order to investigate the impact of CPB on the development of intraoperative hemolysis, we studied changes of fHb, haptoglobin (Hp, the physiologic intravascular fHb scavenger), plasma NO-consumption, and markers of renal tubular damage and intestinal mucosal injury in three groups of patients undergoing cardiac surgery associated with increasing perfusion times: off-pump coronary artery bypass grafting CABG (OPCAB), CABG with CPB, and combined CABG and valve surgery with CPB (CABG+Valve). Also, the incidence of clinical acute kidney injury was evaluated using the AKIN-criteria (Mehta et al., 2007).

## Subjects and methods

### Patients

Based on a pilot study, power analysis indicated that inclusion of 30 patients per study group enabled detection of statistical significant differences in fHb levels. As we hypothesized that the duration of cardiopulmonary bypass time influences the extent of hemolysis, we included 30 consecutive adult patients undergoing elective on-pump CABG surgery and 30 consecutive patients undergoing elective CABG+Valve surgery (reconstruction and/or replacement of any valve), meeting the inclusion and exclusion criteria. Patients undergoing OPCAB surgery were included to serve as a control group as we expected that hemolysis does not develop in this patient group. However, as OPCAB surgery is infrequently performed at our institution, we included all OPCAB patients meeting the inclusion and exclusion criteria during the inclusion period of the CABG and CABG+Valve patients. This resulted in the final inclusion of 7 OPCAB patients. In total, sixty-seven adult patients undergoing elective cardiac surgery at the Department of Cardiothoracic Surgery of the Maastricht University Medical Center+ between November 2009 and January 2011 were studied. The study was approved by the Institutional Review Board and written informed consent was obtained from every patient prior to surgery. A preoperative estimated glomerular filtration rate (eGFR) < 60 ml/min/1.73 m^2^, indicating chronic kidney disease (CKD) according to the KDOQI guidelines (National Kidney Foundation, 2002) in the absence of information regarding structural and/or functional renal abnormalities, was reason for exclusion as these patients are at increased risk for postoperative AKI, irrespective of other (potential) risk factors. In addition, presence of diabetes was reason for exclusion as these patients display higher baseline levels of N-Acetyl-β-D-glucosaminidase (NAG), one of the studied markers. In addition, presence of chronic hemolytic disease was reason for patient exclusion. The cardiac surgery protocol as performed at our institution has been described in more detail elsewhere (Heijmans et al., 2007).

### Study endpoints

The primary study endpoint was to investigate whether hemolysis, reflected by plasma fHb concentrations, causes a significant increase in intravascular NO-consumption during cardiac surgery, thus impairing NO-bioavailability. Second, we evaluated the extent of visceral tissue injury which develops during OPCAB surgery, CABG surgery and CABG+Valve surgery. In addition, the correlation between hemolysis, increased NO-consumption, visceral tissue damage (indicated by increased urinary and plasma levels of renal and intestinal damage), and postoperative AKI was investigated. Lastly, we investigated changes plasma haptoglobin levels, an important fHb binding protein involved in clearance of fHb.

### Definition of postoperative acute kidney injury

Postoperative AKI was defined according to the AKI Network (AKIN) classification (Mehta et al., 2007). This classification for AKI uses either changes in serum creatinine or urine output from a preset baseline (in our case, preoperative level). As we did not have sufficient reliable data on urine output we used the serum creatinine criteria. Patients were stratified according to three grades of postoperative AKI of increasing severity. Stage 1 AKI was defined as an increase in serum creatinine of more than or equal to 0.3 mg/dl (≥26.4 μmol/l) or increase to more than or equal to 150–200% (1.5- to 2-fold) from the preoperative level. Stage 2 AKI was defined as an increase in serum creatinine to more than 200–300% (>2- to 3-fold) from the preoperative level. Stage 3 AKI was defined as an increase in serum creatinine to more than 300% (>3-fold) from baseline (or serum creatinine of more than or equal to 4.0 mg/dl [≥354 μmol/l] with an acute increase of at least 0.5 mg/dl [44 μmol/l]). Patients requiring (transient) postoperative renal replacement therapy were classified as Stage 3, irrespective of creatinine change.

### Blood sampling, urine sampling, and sample processing

Arterial blood and a fresh spot urine sample were obtained at 8 pre-set perioperative time points; 1, preoperatively, after induction but prior to sternotomy; 2, before start of CPB; 3, end CPB; 4, 15 min after cessation of CPB; 5, 2 h after cessation of CPB; 6, 4 h after cessation of CPB; 7, day 1 postoperatively; 8, day 2 postoperatively. Whole blood was collected in EDTA vacutainers (Becton Dickinson, Franklin Lakes, NJ). All samples were immediately put on ice and centrifuged within 15 min after collection (1500 g at 4°C for 15 min without brake), aliquoted, and stored at −80°C until further analysis.

### Analysis of hemolysis, haptoglobin, renal tubular damage, and intestinal damage

Plasma fHb concentrations were measured by derivative spectrometry as previously described (Cruz-Landeira et al., 2002). The lower detection limit of the assay was 2 μM. The course of plasma Hp, the physiological fHb-scavenger (Kristiansen et al., 2001), during surgery was measured on a validated Beckman LX20 clinical chemistry analyzer (Beckman Coulter, Brea, CA) via a turbidimetric method by the Central Diagnostic Laboratory of the Maastricht University Medical Center. To assess renal tubular damage, urinary NAG concentrations were determined by an enzyme colorimetric assay according to the manufacturer's instructions (HaemoScan, Groningen, The Netherlands) (Dittrich et al., 2000). Results were normalized to urinary creatinine to correct for dilution and expressed as U/mmol creatinine. Intestinal mucosal damage was studied using plasma concentrations of intestinal fatty acid binding protein (IFABP) which were assessed by an in-house human IFABP ELISA with a detection limit of 12.5 pg/mL. IFABP is an early, sensitive, and specific marker of clinically relevant intestinal mucosal damage. Furthermore, we recently demonstrated excellent discriminating value of plasma IFABP levels for the detection of clinically significant intestinal ischemia in patients undergoing open abdominal or thoracoabdominal aortic aneurysm repair (Lieberman et al., 1997; Vermeulen Windsant et al., 2012a). As the extent of hemodilution during surgery differed between the three study groups, impairing optimal comparison of the studied plasma proteins fHb, Hp, and IFABP, we corrected these values for plasma hematocrit at the moment of blood sampling.

### NO consumption assay

To evaluate the NO-consuming capacity of plasma by fHb, we randomly selected 29 patients (5 OPCAB, 12 CABG, and 12 CABG+Valve patients) using SPSS. The complete NO consumption protocol is described elsewhere (Reiter et al., 2002; Rother et al., 2005). Briefly, a 40 μM solution of the NO-donor, DETA NONOate (Cayman Chemical, Ann Harbor, MI) was prepared in PBS (pH 7.4) in a glass vessel purged with nitrogen in-line with a NO chemiluminescence analyzer (Sievers Model 280i, GE, Boulder, CO). The subsequent decay of DETA NONOate, releasing NO, produced a steady state NO signal of about 50–70 mV. When the signal became stable, 50 μL of plasma was injected into the DETA NONOate solution, decreasing the NO signal in case of NO-consumption. Data were analyzed with the software program ORIGIN Version 6.1 (OriginLab, Northampton, MA) for analysis of the area under the curve (AUC) of decreasing NO-signal over time. The amount of NO consumption by plasma was quantified by comparison of the AUC with that of NO gas standards (produced from injections of nitrite into triiodide).

### Statistics

Continuous data are presented as median and interquartile range (IQR, 25–75th percentile) or mean ± s.e.m., depending on Gaussian distribution (checked using histograms and normal Q-Q plots). Dichotomous data are depicted as % (N). Differences between study groups were compared using Pearson Chi-square test with Fisher's correction when appropriate (dichotomous variables), or independent sample *T*-test or Kruskal-Wallis test (for continuous variables). Overall changes in fHb, NO-consumption, Hp, NAG, and IFABP levels within groups were tested using the Friedman test for repeated data. If this test yielded a significant result, a Wilcoxon signed ranks test with Bonferroni's correction was used for the *post-hoc* analysis. To characterize total release of fHb, plasma NO- consumption, urinary NAG, and plasma IFABP, the area under the curve (AUC_fHb_, AUC_NO_, AUC_NAG_, and AUC_IFABP_) was calculated for each patient using trapezoidal analysis with time as a baseline. Univariate correlations were Spearman correlations (R_s_) and linear regression analysis (*r*^2^). Multivariable linear regression analysis (“enter method”) enabled correction for other confounding risk factors. Confounding variables known to be associated with increased NAG concentrations and IFABP concentrations were chosen. Statistical calculations were made using SPSS 15.0 (SPSS, Inc., Chicago, IL), and Prism 4.03 (GraphPad Software Inc. San Diego, CA). Values of *p* < 0.05 were considered to be statistically significant.

## Results

### Patient characteristics and outcome

Patients were predominantly male (*N* = 57, 85.1%) and middle-aged (mean 66.5 years, range 38–82 years). Baseline demographic patient data did not significantly differ among patients undergoing OPCAB, CABG, or CABG+Valve surgery (Supplementary Table 1). Preoperative renal function was significantly lower in the latter two patient groups (*p* = 0.003). In total, 9 patients developed AKI (13.4%) of whom 8 patients developed stage 1 AKI, and 1 patient stage 2 AKI. Renal function recovered in all AKI patients prior to discharge and none required (temporary) renal replacement therapy. Serious gastro-intestinal complications such as bowel ischemia did not occur. Two patients (3.0%) developed a postoperative ileus which was successfully, non-surgically, treated. Overall in-hospital mortality was 1.5% (*N* = 1) and cause of death was major neurological injury with subsequent respiratory failure.

### CPB-assisted surgery causes acute hemolysis, increased plasma NO-consumption, and depletion of plasma haptoglobin

**Figure 1:**
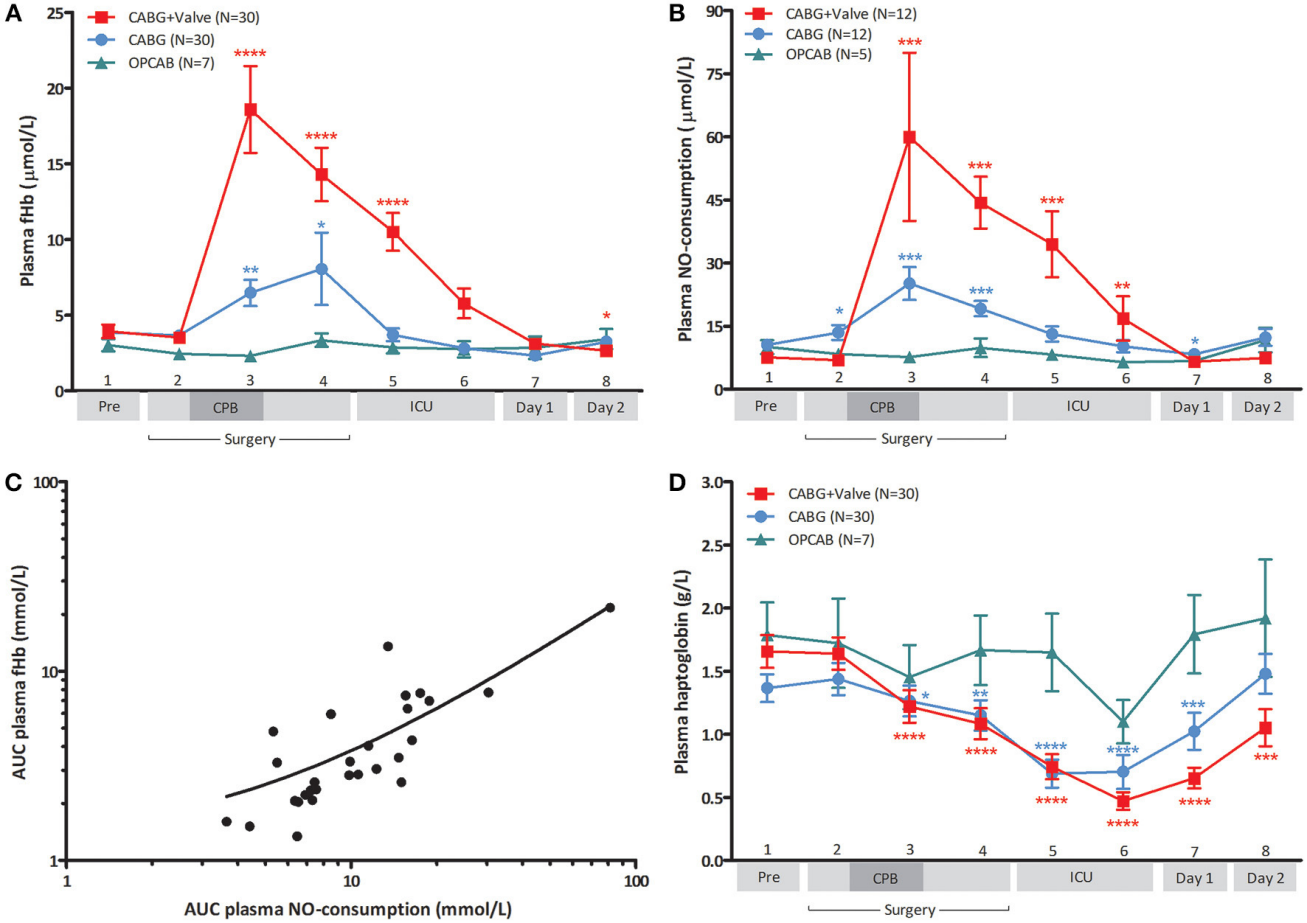
**Change of plasma free hemoglobin (fHb, A), NO-consumption (B), and haptoglobin (D) in patients undergoing CABG+Valve (■ red line), CABG (• blue line), and OPCAB surgery (▲ green line), and correlation between hemolysis and NO-consumption (C)**. The total release of fHb and change in NO-consumption **(C)** during surgery, estimated using the area under the curve (AUC) were significantly correlated (*r*^2^ = 0.75, *p* < 0.0001). **(D)** Depicts the change in perioperative Hp levels in all groups. All values were corrected for hematocrit because of significant intraoperative hemodilution. Values are mean ± s.e.m. **(A,B,D)** or a scatter plot with mean (black line) ± 95% confidence interval (dotted line, **C**). Stars indicate significant changes compared to baseline within groups: ^*^p < 0.05, ^**^p < 0.01, ^***^p < 0.001, ^****^p < 0.0001. Numbers 1–8 on the y-axis **(A,B,D)** refer to collection time-points of blood specimens: 1, preoperatively, after induction but prior to sternotomy; 2, before start of CPB; 3, end CPB; 4, 15 min after cessation of CPB; 5, 2 h after cessation of CPB; 6, 4 h after cessation of CPB; 7, day 1 postoperatively; 8, day 2 postoperatively. CPB was not used in OPCAB patients.

Baseline fHb levels were comparable between the three patient groups (*p* = 0.78, Figure 1A). During surgery, plasma fHb levels increased significantly in patients subjected to CPB (*p* < 0.0001 for CABG, and CABG+Valve surgery patients), peaking at the end of CPB (6.5 ± 0.9 μmol/L, *p* = 0.041 compared to baseline for CABG patients, and 18.6 ± 2.9 μmol/L, *p* < 0.0001, for CABG+Valve patients). After cessation of CPB, plasma fHb concentrations gradually decreased over time in these patient groups. Both peak fHb levels and total release of fHb (AUC_fHb_) was significantly correlated to CPB-duration (*R_s_* = 0.50, *p* < 0.001, and *R*_s_ = 0.55, *p* < 0.001, respectively). Hemolysis did not occur in OPCAB patients (change of plasma fHb over time: *p* = 0.22). The kinetics of plasma NO-consumption was similar to that of plasma fHb in all study groups (Figure 1B). Plasma NO-consumption only significantly increased in patients subjected to on-pump surgery (*p* < 0.001 for CABG and CABG+Valve patients), while baseline NO-consumption was statistically equal between the study groups (*p* = 0.18). The NO-consuming capacity of plasma peaked at the end of CPB, having increased 2.4 fold in CABG patients, and 7.9 fold in CABG+Valve surgery patients. Consistent with the potent NO-scavenging property of fHb, the AUC_fHb_ correlated significantly to total plasma NO-consumption (AUC_NO_, *r*^2^ = 0.75, *p* < 0.0001, Figure 1C).

Physiologically, the plasma protein Hp acts as a buffer against increases in plasma fHb levels through the formation of Hp-fHb complexes resulting in fHb clearance from the circulation (Kristiansen et al., 2001). This way, excessive hemolysis can potentially result in a significant reduction of Hp bioavailability (Tabbara, 1992). Indeed, plasma Hp dropped significantly over time in CABG and CABG+Valve surgery patients (*p* < 0.0001, for both groups, Figure 1D) suggestive of Hp-fHb complex clearance. Loss of circulating Hp was most marked in CABG+Valve surgery patients compared to CABG patients, which related to the higher plasma fHb levels in this patient group. The first statistically significant decrease in plasma Hp was measured at the end of CPB, and concentrations further declined during the postoperative period reaching lowest levels 2 and 4 h after cessation of CPB in CABG and CABG+Valve surgery patients (0.69 ± 0.11 g/L, *p* < 0.0001, and 0.47 ± 0.07 g/L, *p* < 0.0001, respectively). The maximum increase of plasma fHb correlated significantly to the maximum decrease of Hp (*r*^2^ = 0.28, *p* < 0.0001, data not shown). Similar to fHb and NO-consumption, Hp levels remained relatively constant in OPCAB patients throughout surgery (*p* = 0.059). The observed increase of Hp after reaching lowest levels 4 h after cessation of CPB was suggestive of enhanced Hp production and subsequent release of Hp into the circulation.

### CPB-induced hemolysis is associated with visceral injury and clinical acute kidney injury

**Figure 2:**
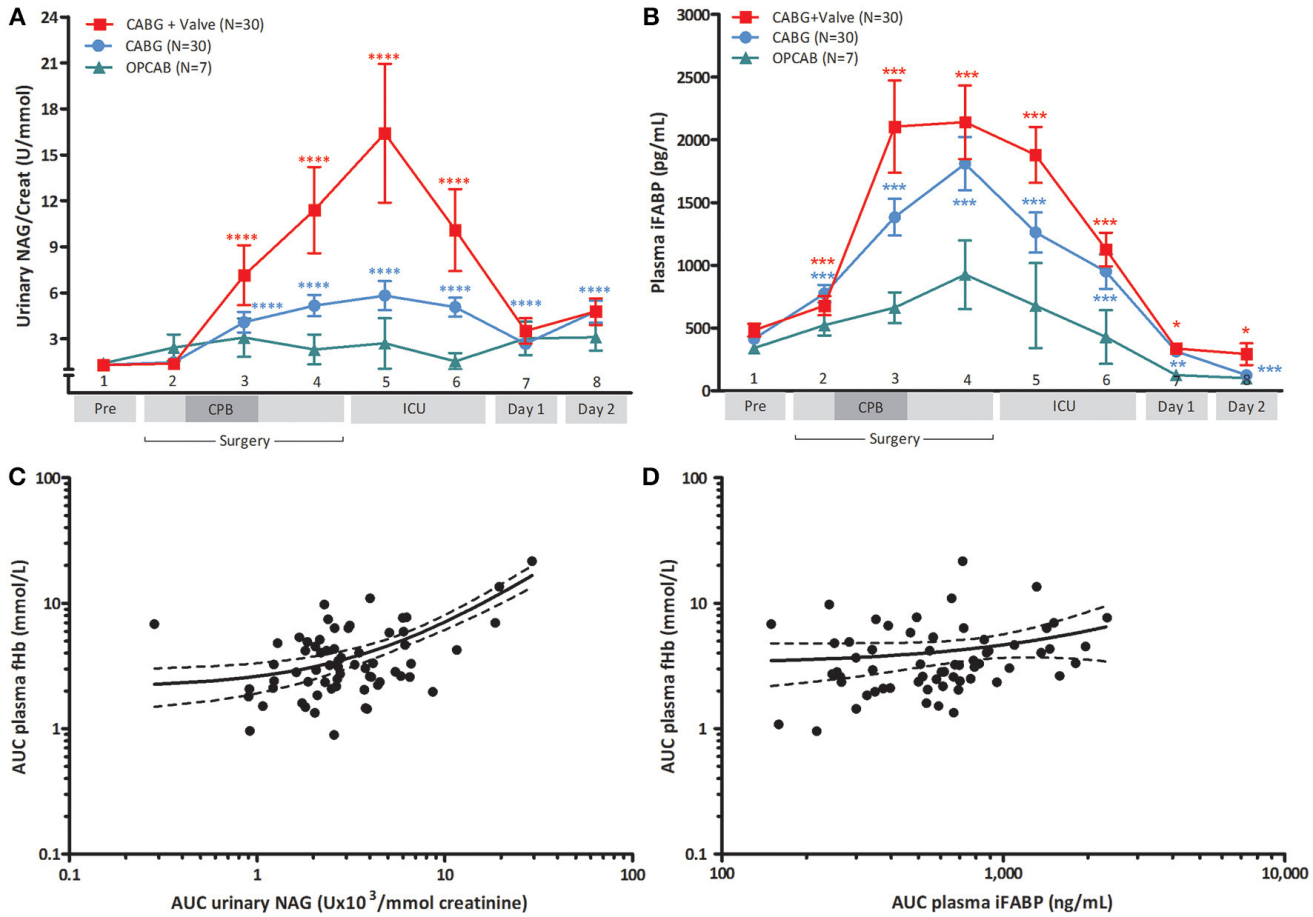
**Change of urinary NAG (A) and plasma IFABP (B) in patients undergoing CABG+Valve (■ red line), CABG (• blue line), and OPCAB surgery (▲ green line), and correlation between hemolysis and NAG (C) or IFABP release (D)**. The total release of fHb and NAG **(C)**, and fHb and IFABP **(D)**, estimated using the area under the curve (AUC), were significantly correlated (*R*_s_ = 0.70, *p* < 0.0001, and *R*_s_ = 0.26, *p* = 0.04, respectively). Plasma IFABP values were corrected for hematocrit because of significant intraoperative hemodilution, urinary NAG levels were corrected for urine creatinine values. Values are mean ± s.e.m. **(A,B)** or a scatter plot with mean (black line) ± 95% confidence interval (dotted line, **C,D**). Stars indicate significant changes compared to baseline within groups: ^*^p < 0.05, ^**^p < 0.01, ^***^p < 0.001, ^****^p < 0.0001. Numbers 1–8 on the y-axis **(A,B)** refer to collection time-points of blood specimens: 1, preoperatively, after induction but prior to sternotomy; 2, before start of CPB; 3, end CPB; 4, 15 min after cessation of CPB; 5, 2 h after cessation of CPB; 6, 4 h after cessation of CPB; 7, day 1 postoperatively; 8, day 2 postoperatively. CPB was not used in OPCAB patients.

The development of renal tubular injury and intestinal mucosal injury was studied using urinary NAG (Figure 2A) and plasma IFABP (Figure 2B). Both parameters were similar in all groups at baseline (*p* = 0.859, and *p* = 0.697, respectively), and increased significantly during CABG and CABG+Valve surgery (*p* < 0.0001, and *p* < 0.001 for NAG and IFABP, respectively). OPCAB patients did not display significant changes of NAG or IFABP over time (*p* = 0.235 and *p* = 0.062, respectively). The potential relation between acute hemolysis and visceral organ injury in patients undergoing cardiac surgery was studied by correlation of AUC_fHb_, AUC_NAG_, and AUC_IFABP_ (Figures 2C,D). Both renal injury and intestinal damage were significantly correlated with the extent of hemolysis during surgery (*R*_s_ = 0.70, *p* < 0.0001, and *R_s_*= 0.26, *p* = 0.04, respectively), albeit this correlation was stronger for renal tubular injury. Importantly, the correlation between hemolysis, renal tubular damage, and intestinal injury remained statistically significant after correction for four other potential confounders using multivariable logistic regression (Tables 1, 2, respectively). The correlation between NO-consumption and both tissue injury markers was comparable to their correlation with fHb (*R*_s_ = 0.55, *p* = 0.002, and *R*_s_ = 0.41, *p* = 0.03, for NAG and IFABP respectively, data not shown), suggestive of a causal role of fHb-induced NO-scavenging in the development of visceral injury during cardiac surgery.

**Table 1:** **Multivariable linear regression for AUC_NAG_**.

**Variable**	**Beta**	**95% CI**	***p* Value**
Age (years)	−0.04	−122.14–84.49	0.72
Preoperative eGFR (ml/min/1.73m^2^)	0.01	−72.26–76.18	0.96
Duration of CPB (min)	−0.08	−21.82–10.19	0.47
Need for packed red blood cell transfusion (yes)	0.12	−1200.34–3530.49	0.33
AUC_fHb_	0.77	0.70–1.32	<0.0001

**Table 2:** **Multivariable linear regression for AUC_iFABP_**.

**Variable**	**Beta**	**Confidence interval (95%)**	***p* Value**
Age (years)	0.16	−5763.55–191117.38	0.28
Cardiac Ejection Fraction (%)	0.24	−86399.79–544968.51	0.15
Intraoperative blood loss (mL)	−0.03	−401.87–339.21	0.87
Number of blood transfusions (units)	−0.15	−182970.49–78106.15	0.42
AUC_fHb_	0.30	0.60–120.77	0.04

**Figure 3:**
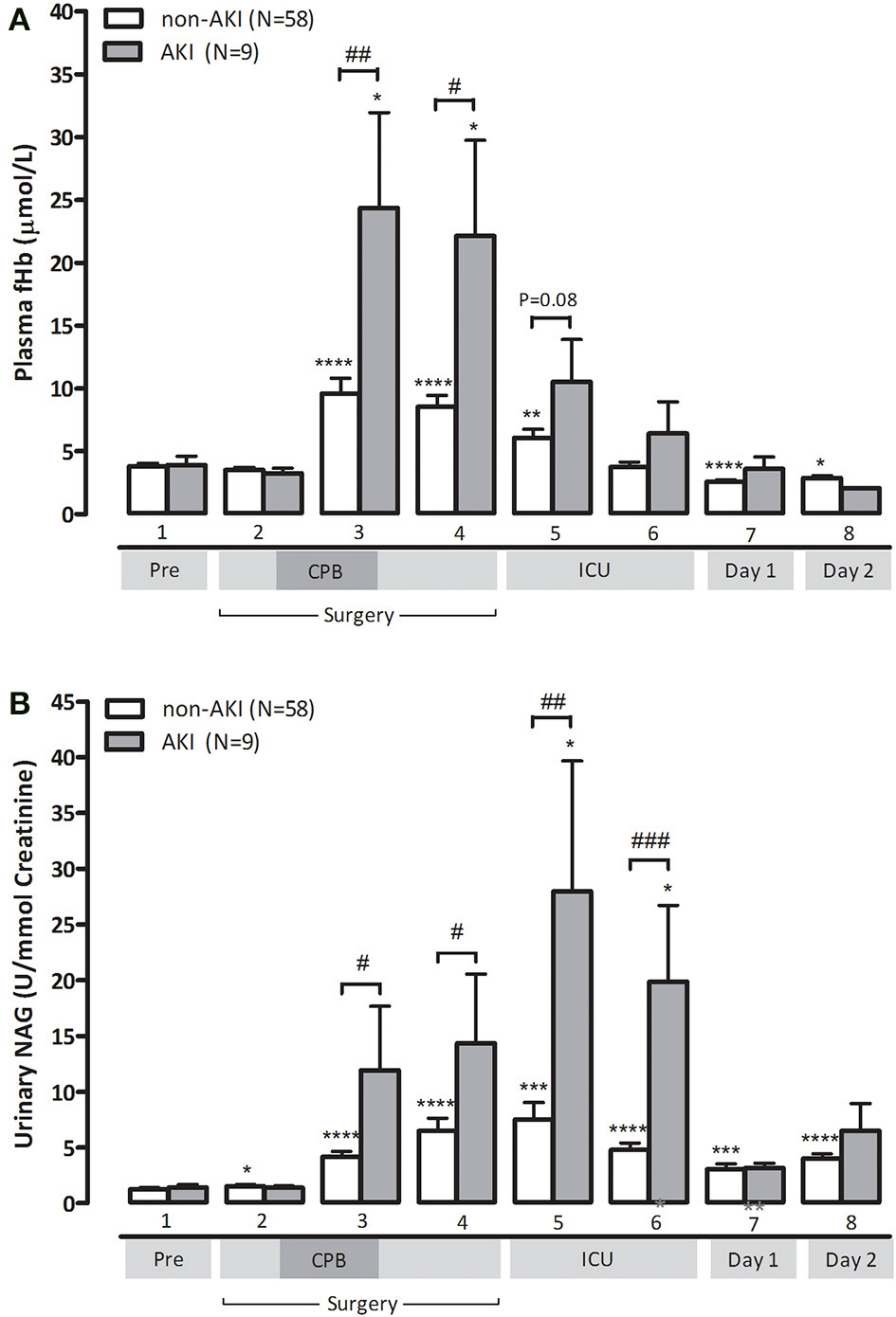
**Patients with postoperative AKI (gray bars) display significantly higher fHb (A) and urinary NAG levels (B), compared to non-AKI patients (white bars)**. Values are mean + s.e.m. Stars indicate significant changes compared to baseline within groups: ^*^p < 0.05,^**^p < 0.01, ^***^p < 0.001, ^****^p < 0.0001. Hash symbols indicate significant differences between groups: ^#^*p* < 0.05, ^##^*p* < 0.01, ^###^*p* < 0.001. Numbers 1–8 on the y-axis refer to collection time-points of blood specimens: 1, preoperatively, after induction but prior to sternotomy; 2, before start of CPB; 3, end CPB; 4, 15 min after cessation of CPB; 5, 2 h after cessation of CPB; 6, 4 h after cessation of CPB; 7, day 1 postoperatively; 8, day 2 postoperatively.

Finally, we studied the relation between hemolysis and development of AKI as defined by an increase in serum creatinine = 150% compared to preoperative values within 48 h after surgery (Mehta et al., 2007). Clinical characteristics of non-AKI and AKI patients are depicted in Supplementary Table 2. Patients developing postoperative AKI were already identifiable during surgery as they displayed significantly higher fHb levels at the end of CPB compared to non-AKI patients (24.4 ± 7.6 vs. 9.6 ± 1.2 μmol/L, *p* < 0.01, Figure 3A). AKI patients also showed significantly higher NAG levels at the end of CPB, indicating more profound renal tubular injury compared to non-AKI patients (11.9 ± 5.7 U/mmol vs. 4.2 ± 0.5 U/mmol, *p* < 0.05, Figure 3B). We could not analyze NO-consumption in AKI and non-AKI patients due to small patient numbers as NO-consumption was only assessed in a (random) selection of patients.

## Discussion

In the current study we are the first to demonstrate that hemolysis occurring during cardiothoracic surgery strongly correlates to increased plasma NO-consumption. Moreover, hemolysis is correlated to the development of intestinal mucosal injury and AKI. Patients subjected to more complex types of cardiac surgery, such as combined bypass and cardiac valve surgery, appear to be at highest risk.

The adverse effect of hemolysis on the intravascular NO-metabolism is increasingly appreciated as an important pathophysiological mechanism in the development of complications in various clinical settings. The discovery of oxygenated plasma fHb (HbFe^2+^O_2_) as a potent scavenger of endothelial-derived NO has provided an explanation for the development of gastro-intestinal smooth muscle dystonias, vasculopathy, endothelial dysfunction, renal injury, pulmonary hypertension, and increased coagulation in patients with chronic hemolytic disease, patients undergoing hemodialysis and increased mortality in septic patients (Reiter et al., 2002; Rother et al., 2005). In the present study, acutely enhanced fHb concentrations during surgery caused a 2.4-fold increase in plasma NO-consumption in CABG patients (compared to preoperative levels), and a 7.9-fold increase in CABG+Valve patients, in whom plasma NO-consumption levels of 266 μmol/L were observed. As plasma NO-consumption levels of approximately 10–15 μmol/L already have been related to a significant impairment in NO-dependent fore-arm blood flow response in patients, reflective of impaired (microcirculatory) perfusion, the extent of NO-consumption measured in these patients is of clinical significance (Reiter et al., 2002; Meyer et al., 2010). Such a decrease in NO-bioavailability promotes vasoconstriction, platelet aggregation, up regulation of adhesion molecule expression, and stimulation of vascular leucocyte adhesion and inflammation (Reiter et al., 2002).

Hemolysis is a well-recognized consequence of CPB, and has been shown to increase with longer perfusion times. (Cheung et al., 2007; Vercaemst, 2008). Three factors principally contribute to the development of CPB-induced hemolysis: (1) exposure of RBCs to mechanical forces, particularly shear stress(Vercaemst, 2008), (2) contact of blood with air or non-endothelial surfaces (Dejam et al., 2007), and (3) use of suction pressures (Gregoretti, 1996). In addition, the development of sublethal RBC damage, promoting premature intravascular RBC disintegration and altering the rheological properties of blood, further contribute to increased fHb levels during surgery (Watanabe et al., 2007). Furthermore, autotransfusion devices and transfusion of stored red blood cells may contribute to increased fHb levels during cardiac surgery by causing significant sublethal damage to red blood cells, making them prone to premature intravascular lysis (Vercaemst, 2008; Kim-Shapiro et al., 2011; Vermeulen Windsant et al., 2012b). In line, we measured highest fHb levels in CABG+Valve surgery patients, exposed to the longest CPB times, and requiring the highest number of stored red blood cell transfusions. This may be further explained by increased red blood cell trauma induced by active pericardial suction. In contrast, OPCAB patients did not display significant changes of plasma fHb over time.

As the development of hemolysis during CPB is well known, there have been several, albeit a few, studies reporting on the adverse relation between intraoperative hemolysis and patient outcome after cardiovascular surgery. These studies all focused on the direct toxic effect of fHb on the renal epithelium by fHb-induced generation of damaging reactive oxygen species (catalyzed by free heme and iron), and formation of obstructive casts (Zager and Gamelin, 1989; Tanaka et al., 1991; Davis et al., 1999; Baumgart and Dignass, 2002; Haase et al., 2007). As fHb was believed to be merely nephrotoxic after glomerular filtration, a correlation between hemolysis and injury of other organs in the context of CPB has, to our knowledge, never been reported. The additional NO-scavenging property of fHb, next to its nephrotoxic effect, could however explain the stronger correlation between hemolysis and renal injury compared to the correlation between hemolysis and intestinal injury in this study. The fact that in this setting hemolysis correlates with injury of two independent organ systems has significant implications for the (potential) harmful effect of fHb on other tissues. It has been reported that fHb impairs left ventricular function and coronary blood flow in neonatal rabbit hearts, particularly following ischemia and reperfusion (Nemeto et al., 2000). Furthermore, the correlation between hemolysis and pulmonary hypertension in the setting of chronic hemolysis is well established (Gladwin et al., 2004).

The results of this study offer a window of opportunity to reduce surgery related morbidity and mortality, particularly in high risk patients. Inactivation of fHb or increasing NO-bioavailability may maintain vascular homeostasis, stimulate microcirculatory blood flow, and thus limit tissue injury during CPB-assisted cardiac surgery. Four therapeutic options are of particular interest in the current setting. First, prophylactic and/or therapeutical administration of (recombinant) Hp could counteract the depletion of Hp we observed during, and after surgery. In fact, administration of Hp significantly reduced urinary NAG levels and prevented hemoglobinuria in 14 patients undergoing CABG surgery with CPB (Tanaka et al., 1991). Unfortunately, high costs currently limit clinical implementation of routine Hp supplementation, though plasma transfusion could be an option. Second, administration of NO-gas via the oxygenator or ventilation gas, analogous to inhaled NO (iNO), may prove to be beneficial. INO, already at very low concentrations (80 ppm), inactivates fHb in the pulmonary vasculature through oxidation, and reduces the NO-consuming capacity of plasma (Reiter et al., 2002). INO enhances the formation of NO-metabolites, such as nitrate and nitrite, which are important for intravascular NO-transport and cytoprotection (Lundberg et al., 2008). Also, iNO decreases shear stress induced sublethal RBC damage (Baskurt et al., 2004). Inhaled NO is already clinically used in various settings (Bloch et al., 2007), and it neither alters NO-consumption in healthy volunteers nor results in significant methemoglobinemia (Reiter et al., 2002). Third, the direct oral or intravenous administration of nitrite or nitrate to increase the plasma NO-donor pool could be worth investigating (Lundberg et al., 2008). Finally, the administration of drugs directly activating or stimulating soluble guanylate cyclase, promoting vasodilation independent of endogenous NO, may be a promising new approach (Raat et al., 2013).

The main strength of our study is the inclusion of patients undergoing different types of cardiac surgery of various complexity. This allowed identification of patients at highest risk for hemolysis-induced organ injury; those who may benefit from hemolysis-targeted therapeutic interventions. Furthermore, the exclusion of patients with preoperative CKD limited confounding on the outcome measures renal tubular injury and postoperative AKI. Nevertheless, we cannot exclude presence of preoperative CKD with mildly impaired or preserved renal function (stage 1 and 2 CKD according to the KDOQI classification, 2002) as a confounding factor, as we did not have information on the presence of structural or functional renal damage. The main limitations are inherent to the observational nature of the study. First, it is not possible to determine causation from the presented data. Second, the number of adverse clinical events (AKI) was relatively low, and thus precludes firm conclusions as to the impact of hemolysis on adverse clinical outcome. Furthermore, no patient developed clinical transmural intestinal necrosis, hence a clear correlation between hemolysis and clinical intestinal injury could not be made. Nevertheless, the significant increase in plasma IFABP levels (and its correlation to increased fHb concentrations) reflects mucosal intestinal damage, which has been clearly associated with promotion of a pro-inflammatory response syndrome and adverse clinical outcome (Hanssen et al., 2008; Grootjans et al., 2010). Third, it cannot be excluded that increased hemolysis and NO-consumption are a consequence of increased morbidity rather than a mediator. Finally, other potential mechanisms underlying the relation between intravascular hemolysis and kidney damage, such as heme induced oxidative damage were not investigated and may be important in this setting (Baumgart and Dignass, 2002).

In conclusion, this study demonstrates a significant adverse relation between enhanced plasma fHb levels, decreased plasma NO-bioavailability, AKI, and organ integrity loss during cardiac surgery. Patients undergoing complex cardiac surgery, such as combined valve and CABG surgery, appear to be at highest risk for hemolysis-induced organ injury, probably potentiated by long perfusion times and high transfusion requirements. These results call for increased awareness for the adverse consequences of even modest increases in plasma fHb during cardiac surgery and additional studies are needed to investigate whether inactivation of fHb or abrogation of its effects on the (micro)circulation significantly attenuates the development of visceral tissue injury and AKI, thus improving patient outcome after CPB-assisted cardiac surgery.

## Author contributions

Iris C. Vermeulen Windsant: principal contribution to design of the work, acquisition, analysis, and interpretation of data. Principal drafting, revising, and (final) approval of the manuscript. Accountable for all aspects of the work. Norbert C. J. de Wit and Jose E. Tanus-Santos: important contribution to design of the work, analysis, and interpretation of data. Contributed to drafting, revising, and (final) approval of the manuscript. Accountable for all aspects of the work. Jonas T. C. Sertorio and Annemarie A. van Bijnen: contributed to design of the work, and analysis of data. Contributed to drafting, revising, and (final) approval of the manuscript. Accountable for all aspects of the work. Yuri M. Ganushchak and John H. Heijmans: contributed to design of the work, and interpretation of data. Contributed to drafting, revising, and (final) approval of the manuscript. Accountable for all aspects of the work. Michael J. Jacobs and Jos G. Maessen: principal contribution to design of the work, and interpretation of data. Contributed to drafting, revising, and (final) approval of the manuscript. Accountable for all aspects of the work. Wim A. Buurman: principal contribution to design of the work, analysis, and interpretation of data. Principal drafting, revising, and (final) approval of the manuscript. Accountable for all aspects of the work.

### Conflict of interest statement

The authors declare that the research was conducted in the absence of any commercial or financial relationships that could be construed as a potential conflict of interest.
