# Supplementary material for: Hemolysis during cardiac surgery is associated with increased intravascular nitric oxide consumption and perioperative kidney and intestinal tissue damage
Source: Front Physiol. 2014 Sep 8;5:340. doi: 10.3389/fphys.2014.00340 (PMC4157603; doi:10.3389/fphys.2014.00340)
Supplement: Supplementary file 1 [file DataSheet1.ZIP › Supplementary Table 1.DOCX]

**Supplementary Table 1 Perioperative patient characteristics**

| **Characteristics** | **OPCAB (N=7)** | **CABG (N=30)** | **CABG + Valve (N=30)** | **p-value** |
| --- | --- | --- | --- | --- |
| Male | 71.4 (5) | 90.0 (27) | 83.3 (25) | 0.367 |
| Age (years) | 64 (39-67) | 71 (60-73) | 72 (62-77) | 0.054 |
| BMI (kg/m^2^) | 25.0 (23.5-26.3) | 27.7 (25.5-31.0) | 26.5 (26.3-30.6) | 0.138 |
| Hypertension (yes) | 57.1 (4) | 90.0 (27) | 83.3 (25) | 0.067 |
| Currently smoking (yes) | 0.0 (0) | 16.7 (5) | 16.7 (5) | 0.631 |
| Hypercholesterolemia (yes) | 57.1 (4) | 86.7 (26) | 86.7 (26) | 0.074 |
| Myocardial infarction (yes) | 14.3 (1) | 40.0 (12) | 26.7 (8) | 0.283 |
| PAOD (yes) | 28.6 (2) | 16.7 (5) | 10.0 (3) | 0.078 |
| COPD (yes) | 0.0 (0) | 16.7 (5) | 13.3 (4) | 0.596 |
| Stroke (yes) | 14.3 (1) | 6.7 (2) | 13.3 (4) | 0.614 |
| Ejection fraction < 50% (yes) | 28.6 (2) | 30.0 (9) | 43.3 (13) | 0.116 |
| Serum creatinine (μmol/L) | 75 (69-78) | 85 (78-91) | 87 (77-97) | 0.012 |
| Estimated GFR (ml/min/1.73m^2^) | 88 (86-102) | 76 (71-86) | 71 (64-85) | 0.003^×^ |
|  |  |  |  |  |
| Duration of surgery (min) | 120 (108-179) | 191 (158-240) | 233 (188-263) | <0.001^§^ |
| ACC time (min) | - | 40 (35-70) | 106 (73-122) | <0.001^#^ |
| Duration of CPB (min) | - | 68 (55-112) | 145 (101-160) | <0.001^#^ |
| Transfused pRBCs (units) | 0.0 (0) | 1.5 (1.0-2.0) | 2.0 (1.5-4.0) | <0.001^§^ |
| Use of pericardial suction (yes) | 0.0 (0) | 3.3 (1) | 100 (30) | <0.001^§^ |
| Urine production (ml) | 300 (92-720) | 443 (264-593) | 360 (273-493) | 0.454 |
| Blood loss during surgery (ml) | 175 (150-300) | 200 (120-300) | 300 (190-530) | 0.122 |
|  |  |  |  |  |
| In hospital mortality | 0 (0) | 0 (0) | 3.3 (1) | 0.545 |
| Increase of serum creatinine < 48h compared to preoperative value (%) | 103 (94-109) | 109 (96-118) | 114 (101-125) | 0.160 |
| Acute kidney injury (yes) |  |  |  | 0.603 |
| Stage 0 (no AKI) | 100.0 (7) | 90. 0(27) | 80.0 (24) |  |
| Stage 1 | 0.0 (0) | 10.0 (3) | 16.7 (5) |  |
| Stage 2 | 0.0 (0) | 0.0 (0) | 3.3 (1) |  |
| Stage 3 | 0.0 (0) | 0.0 (0) | 0.0 (0) |  |
| Duration of ICU admittance (days) | 1.0 (1.0-1.0) | 1.0 (1.0-1.0) | 1.0 (1.0-2.0) | 0.090 |
| Duration of hospitalization (days) | 6.0 (6.0-8.0) | 7.0 (6.8-9.0) | 7.0 (6.5-13.0) | 0.148 |

**Values are % (N) or median (25^th^-75^th^ IQR);* ^×^*CABG + Valve and CABG versus OPCAB;* ^§^*CABG + Valve versus CABG and OPCAB;* ^#^CABG + Valve versus CABG; **Abbreviations: OPCAB = off-pump coronary artery bypass grafting; CABG = on-pump coronary artery bypass grafting; CABG + Valve = on-pump coronary artery bypass grafting and any type of valve reconstruction or replacement; PAOD = peripheral arterial occlusive disease; COPD = chronic obstructive pulmonary disease; GFR = glomerular filtration rate; ACC = aortic cross clamp; CPB = cardiopulmonary bypass; ICU = intensive care unit*
