# Supplementary material for: Hemolysis during cardiac surgery is associated with increased intravascular nitric oxide consumption and perioperative kidney and intestinal tissue damage
Source: Front Physiol. 2014 Sep 8;5:340. doi: 10.3389/fphys.2014.00340 (PMC4157603; doi:10.3389/fphys.2014.00340)
Supplement: Supplementary file 1 [file DataSheet1.ZIP › Supplementary Table 2.DOCX]

**Supplementary Table 2 Clinical characteristics of non-AKI and AKI patients**

| **Characteristics** | **Non-AKI (N=58)** | **AKI (N=9)** | **p-value** |
| --- | --- | --- | --- |
| Type of surgery |  |  | 0.176 |
| OPCAB | 12.1 (7)* | 0.0 (0) |  |
| CABG | 46.6 (27) | 33.3 (3) |  |
| CABG + Valve | 41.4 (24) | 66.7 (6) |  |
| Male | 86.2 (50) | 77.8 (7) | 0.614 |
| Age (years) | 68 (57 – 73) | 73 (66 – 77) | 0.107 |
| Ejection fraction < 50% (yes) | 23.1 (12) | 33.3 (3) | 0.119 |
| Hypertension (yes) | 81.0 (47) | 100 (9) | 0.334 |
| PAOD (yes) | 13.8 (8) | 22.2 (2) | 0.465 |
| Preoperative serum creatinine (μmol/L) | 83 (77 – 94) | 83 (75 – 96) | 0.797 |
| Preoperative estimated GFR (ml/min/1.73m^2^) | 77 (69 – 86) | 80 (62 – 92) | 0.659 |
|  |  |  |  |
| Duration of surgery (min) | 195 (160 – 240) | 245 (181 – 270) | 0.147 |
| ACC time (min) | 69 (39 – 106) | 78 (53 – 120) | 0.255 |
| Duration of CPB (min) | 105 (63 – 148) | 118 (79 – 159) | 0.431 |
| Transfusion of pRBCs (yes) | 25.9 (15) | 6 (66.7) | 0.010 |
| Use of pericardial suction (yes) | 41.4 (24) | 77.8 (7) | 0.070 |
| Urine production during surgery (ml) | 388 (263 – 568) | 415 (331 – 496) | 0.800 |
| Blood loss (ml) | 245 (150 – 360) | 200 (188 – 750) | 0.542 |
|  |  |  |  |
| In-hospital mortality | 1.7 (1) | 0.0 (0) | 1.000 |
| Duration of ICU admittance (days) | 1 (1 – 1) | 1 (1 – 5) | 0.111 |
| Duration of hospitalization (days) | 7 (6 – 8) | 9 (8 – 14) | 0.007 |

** Values are % (N) or median (25th-75th IQR). Abbreviations: OPCAB = off-pump coronary artery bypass grafting; CABG = on-pump coronary artery bypass grafting; CABG+Valve = on-pump coronary artery bypass grafting and any type of valve reconstruction or replacement; PAOD = peripheral arterial occlusive disease; GFR = glomerular filtration rate; ACC = aortic cross clamp; CPB = cardiopulmonary bypass; pRBC = packed red blood cells; ICU = intensive care unit.*
